# Drug-induced acute pancreatitis in a bodybuilder: a case report

**DOI:** 10.1186/s13256-022-03329-3

**Published:** 2022-03-22

**Authors:** Seyed Ali Safizadeh Shabestari, Samuel B. Ho, Priyadarshini Chaudhary, Rahul A. Nathwani

**Affiliations:** 1 Mohammed Bin Rashid University of Medicine and Health Sciences, Dubai Healthcare City, Building 14, 505055, Dubai, United Arab Emirates; 2 Department of Gastroenterology, Mediclinic City Hospital, North Wing Clinic, Dubai Healthcare City, Building 35, 505004, Dubai, United Arab Emirates; 3 Department of Radiology, Mediclinic City Hospital, Dubai Healthcare City, Building 31, 505004, Dubai, United Arab Emirates

**Keywords:** Drug-induced acute pancreatitis, Anabolic–androgenic steroids, Growth hormone, Clenbuterol, Bodybuilder, Case report

## Abstract

**Background:**

Unregulated use of a variety of drugs and supplements by bodybuilders and athletes is common and can lead to severe adverse complications. Only a small proportion of acute pancreatitis cases are drug induced, and case reports are essential for identifying potential drug-related risks for pancreatitis. Here we present the first case report published of acute pancreatitis linked to recreational use of anabolic–androgenic steroids, subcutaneous growth hormone, and clenbuterol in a previously healthy male after excluding all other causes of pancreatitis.

**Case presentation:**

A 31-year-old Arab male bodybuilder presented with acute abdominal pain associated with nausea and sharp pain radiating to the back. The patient was not using tobacco or alcohol but was using multiple drugs related to bodybuilding, including anabolic–androgenic steroids, subcutaneous growth hormone, clenbuterol, and multiple vitamin supplements. Laboratory studies revealed a normal white blood cell count, elevated C-reactive protein, minimally elevated aspartate aminotransferase and total bilirubin with normal remaining liver tests, and elevated amylase and lipase. The patient had no hypertriglyceridemia or hypercalcemia, and had had no recent infections, abdominal procedures, trauma, or scorpion exposure. Imaging and laboratory investigations were negative for biliary disease and IgG4 disease. Abdominal computed tomography revealed hepatomegaly and diffuse thickening and edema of the body and tail of the pancreas with peripancreatic fat stranding. An abdominal ultrasound showed slight hepatomegaly with no evidence of cholelithiasis. Genetic testing for hereditary pancreatitis-related mutations was negative. A diagnosis of drug-induced acute pancreatitis was made, and he was treated with aggressive intravenous hydration and pain management. The patient has avoided further use of these drugs and supplements and had no further episodes of pancreatitis during 1 year of follow-up.

**Conclusions:**

This case describes a patient with drug-induced acute pancreatitis after the intake of anabolic–androgenic steroids, subcutaneous growth hormone, and clenbuterol, where all other common causes of acute pancreatitis were excluded. Clinicians should be alert to the possibility of drug-induced acute pancreatitis occurring in bodybuilders and athletes using similar drug combinations.

## Introduction

Supplements that enhance athletic performance and aesthetic appearance, such as growth hormone and anabolic–androgenic steroids (AAS), are commonplace among today’s bodybuilder community [1, 2]. The lack of understanding and awareness of the detrimental adverse effects of such drugs can affect athletes’ physical and mental health [2]. The alarming increase in the unregulated use of these substances by bodybuilders can result in a variety of organ-specific pathologies, including pancreatitis, which is an inflammatory process that arises from pancreatic enzymes autodigesting the gland. The worldwide prevalence of acute pancreatitis is not known, but the annual estimates range between 5 and 80 per 100,000 people, with better-recorded data in the USA and Finland [3]. The effects could range from mild to severe, with approximate mortality rates ranging from < 1% to > 30% [4, 5]. The most common presentation is severe epigastric pain, usually radiating to the back, and the diagnosis can be made through a combination of serum amylase and lipase, in addition to imaging studies such as abdominal ultrasound (US) and computed tomography (CT) scans. Gallstones (30–60%) and heavy alcohol use (15–30%) are the most reported causes of acute pancreatitis. However, endoscopic retrograde cholangiopancreatography (ERCP), trauma, hypertriglyceridemia, hyperparathyroidism, pancreatic tumors, surgery, infections, anatomic variants, and drug-induced acute pancreatitis (DIAP) are other less common etiologies.

Due to the inadequate literature on DIAP cases, the exact incidence and prevalence of this condition are not known. The diagnosis is one of exclusion, and immediate management of DIAP is to withdraw the offending agent and provide supportive care. If the offending drug is not identified early, it can result in irreversible damage to the gland, as well as increased length of hospitalization and repeated hospitalizations because of continuous intake of the substance [6, 7]. Hence, case reports are imperative to increasing awareness of uncommon causes of acute pancreatitis.

## Case presentation

A 31-year-old Arab male bodybuilder presented to the emergency department with an acute onset of severe epigastric pain radiating to the back with associated nausea. He did not report any constitutional symptoms of weight loss, fever, chills, fatigue, or cardiovascular, respiratory, neurological, musculoskeletal, hematological, or endocrinological diseases. There was no significant medical or surgical history. There was no known malignancy, infection, trauma, or exposure to scorpions. Family history was insignificant, including for pancreatitis. The patient denied any history of smoking or alcohol consumption. His diet is protein-rich, with high meat consumption. He admitted to starting the following cocktail of drugs a month before: Halotestin (fluoxymesterone), Proviron (mesterolone), Masteron four times per week (drostanolone propionate), Winstrol four times per week (stanozolol), Nolvadex (tamoxifen), “test-E” two times per week (testosterone enanthate), “prop” four times per week (testosterone propionate), “tren” four times per week (trenbolone acetate), “clen” three times per week (clenbuterol), and multiple vitamin supplements. He was also injecting growth hormone five times per week from an inexpensive supplier at double the recommended dose.

**Fig. 1:**
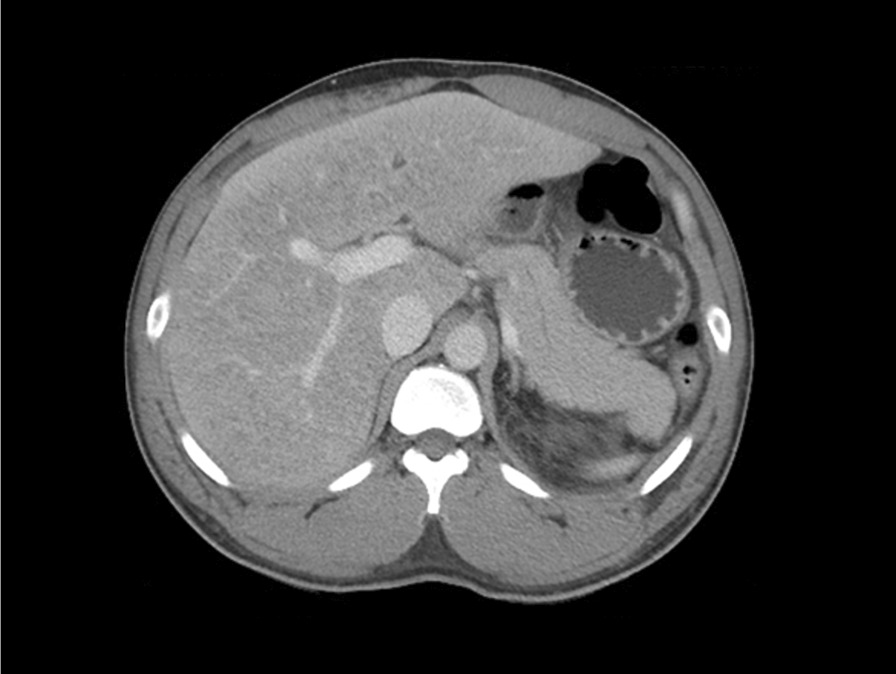
Abdominal computed tomography in the axial plane showing diffuse thickening and edema of the body and tail of the pancreas with peripancreatic fat stranding and no evidence of pancreatic necrosis

On admission, he was hemodynamically stable. His physical examination revealed significant epigastric tenderness without rebound. Laboratory studies revealed slightly low hemoglobin: 12.9 (13.5–17.5) g/dL; normal white blood cell count (WBC): 9.2 × 10^9^ (4.5 to 11.0) × 10^9^/L; elevated C-reactive protein (CRP): 12.9 (13.5–17.5) g/dL; normal troponin, and creatinine: 0.713 (0.2–1.2) mg/dL; elevated creatine protein kinase (CPK): 221 (30–190) U/L; sodium (Na^+^): 137 130–145) mmol/L; potassium (K^+^): 4.57 (3.5–5.4) mmol/L; slightly elevated aspartate aminotransferase (AST): 47 (6–34) U/L; and total bilirubin: 22.70 (3.4–20.50) U/L, with normal remaining liver tests: alanine aminotransferase (ALT): 45 (0–55) U/L; alkaline phosphatase (AP): 33 (1–60) U/L; and elevated amylase and lipase: 525 (30–11) U/L and 503 (23–300) U/L, respectively. An abdominal CT scan with both oral and intravenous contrast revealed hepatomegaly and diffuse thickening and edema of the body and tail of the pancreas with peripancreatic fat stranding but no evidence of fluid collection or pancreatic necrosis (Fig. 1). Based on clinical presentation and CT findings, the patient was diagnosed with acute pancreatitis with Bedside Index of Severity in Acute Pancreatitis (BISAP) score of 0 (< 1% risk of mortality), which is characterized by the absence of organ failure and local or systemic complications.

To identify the cause of his acute pancreatitis, extensive history and workup with additional labs were done, including IgG4 levels, serum triglyceride levels, and serum calcium levels, all of which were normal. A hereditary pancreatitis gene panel revealed no evidence of *CFTR*, *PRSS1*, or *SPINK* gene mutations. He improved clinically with aggressive intravenous hydration and pain management and was discharged after 3 days. An abdominal ultrasound was performed a week later, showing slight hepatomegaly with no evidence of cholelithiasis, and a normal pancreas.

The most likely etiology for his pancreatitis was the mixture of drugs he had started using a month before his presentation, given the exclusion of all other possible causes and the temporal relation between the intake of the substances and the onset of symptoms. Subsequently, he was advised against using any further steroids, growth hormones, or other supplements. He has been symptom-free during a follow-up period of 1 year, with no further evidence of pancreatitis, which is another reason to suspect it was DIAP. Table 1 summarizes our case’s timeline.

**Table 1:** Timeline table

Relevant past medical history and interventions	
Past medical history insignificant for any pancreatic diseases or risk factors. He admitted to using anabolic–androgenic steroids, clenbuterol, multiple vitamin supplements, and growth hormone injection from an inexpensive supplier at double the recommended dose	

Summaries from initial and follow-up visits	Diagnostic testing	Interventions
Based on clinical presentation and CT findings, the patient was diagnosed with acute pancreatitis with Bedside Index of Severity in Acute Pancreatitis (BISAP) score of 0 (< 1% risk of mortality), which is characterized by the absence of organ failure and local or systemic complications. During his hospital stay, the patient was managed with aggressive intravenous hydration and pain management, with significant improvement noticed within 3 days, after which he was discharged home	Laboratory studies: amylase, lipase	Discontinuation of offending drugs (anabolic–androgenic steroids, subcutaneous growth hormone, and clenbuterol); aggressive intravenous hydration and pain management
Patient had no further episodes of pancreatitis during 2 years of follow-up	Imaging: abdominal CT, abdominal ultrasound	

## Discussion

The etiology of acute pancreatitis can be challenging, even after the most common causes have been ruled out. Our patient provided the first four components of the IAP/APA evidence-based guidelines for the initial diagnostic workup of acute pancreatitis: (1) a detailed personal history, (2) a family history, (3) a physical examination, and (4) laboratory tests (that is, liver enzymes, calcium, triglycerides) [4]. Multiple imaging studies, IgG4 testing, and genetic testing eliminated common causes of acute pancreatitis. Also, the elevated creatine protein kinase, slightly elevated AST, and normal troponin were probably due to a skeletal muscle source that is not uncommon among body builders [5]. In addition, the patient may have mild hepatitis secondary to the use of anabolic steroids, and further workup did not reveal any other causes, such as viral, autoimmune, or metabolic causes of hepatitis. However, since the etiology could not be determined after this workup, and the patient admitted to taking AAS, growth hormone, and clenbuterol, we presumed that this was a case of DIAP. Although drug-related causes of acute pancreatitis are rare overall (0.1–2%), numerous drugs have been reported to cause acute pancreatitis [8–11]. As most of the knowledge of DIAP comes from case reports and case series, the actual incidence may be higher [9, 12]. Among the most common are analgesics and antiinflammatory medications, accounting for approximately 30%, followed by antimicrobials and cardiovascular and immunomodulatory drugs (mainly azathioprine and 6-mercaptopurine) [13]. If the symptoms follow drug administration in a close temporal sequence, improve after cessation of the drug, and reappear after repeated exposure, then causality is classified [14]; however, in practice, rechallenge with the putative offending drug is rarely done.

The wide range of drugs implicated as causes of DIAP have been well described [9]. After analysis of these reviews and a comprehensive literature search, we concluded that there have been no previous reports of possible drug-induced acute pancreatitis caused by this type of drug combination or by these individual components: fluoxymesterone, mesterolone, drostanolone propionate, stanozolol, tamoxifen, testosterone enanthate, and testosterone propionate. A literature review of Medline and PubMed Central for case reports, using the search terms “growth hormone,” “pancreatitis,” and “athlete,” yielded only two results, one of which was a case of acute pancreatitis caused by arginine in a 16-year-old athlete [15]. Furthermore, using the search terms “bodybuilder” and “pancreatitis” resulted in only two case reports [16, 17]. After an extensive literature search, we believe that this is the first reported case of possible AAS/clenbuterol/growth-hormone-induced acute pancreatitis. However, there have been previous reports of acute pancreatitis associated with trenbolone acetate [16] and tamoxifen [18] intake. Table 2 compares our patient’s case with published data in the literature related to drug-induced acute pancreatitis in athletes and bodybuilders. It is challenging to perform a causality assessment for the drugs in this patient as he was using multiple bodybuilding supplements concomitantly and stopped them altogether. A causality assessment using the Naranjo scale or the modified scale proposed by Weissman *et al.* [19] should be performed for each potential agent to distinguish between them. There are numerous case reports of gonadal hormones causing acute pancreatitis, and there is a high likelihood that these are causative; however, it may be more likely that the multiple different drugs and drug categories involved in this case play a unique role in causing acute pancreatitis. Meczker *et al.* found in a recent systematic review of 1060 cases of DIAP that gonadal hormones were implicated in 2.36% of all cases of DIAP, whereas multidrug use was implicated in 7.36% [9].

**Table 2:** Cases of acute pancreatitis associated with drugs related to weightlifting or bodybuilding

Case	Patient	Findings	Drug(s) taken	Delay between introduction of the drug and pancreatitis	Rechallenge	Outcome
Safizadeh Shabestari *et al*. 2021 (our case)	31-year-old male	Elevated amylase and lipase, with CT abdomen showing pancreatitis	Fluoxymesterone, mesterolone, drostanolone propionate, stanozolol, tamoxifen, testosterone enanthate, testosterone propionate, clenbuterol, and growth hormone	1 month	Not performed	Clinical improvement with fluid hydration and pain management
Kumar *et al*. [16]	24-year-old male	Elevated amylase and lipase, with CT abdomen showing pancreatitis	Trenbolone acetate	Several months	Not performed	Clinical improvement with conservative treatment
Binet *et al*. [27]	28-year-old male	Isolated elevation of lipase, with CT abdomen showing pancreatitis	l-Arginine alpha-ketoglutarate and other vitamins and supplements	18 months	Not performed	Clinical improvement with conservative treatment
Liane *et al*. 2016 [5]	20-year-old male	Elevated amylase and lipase, LDH, and CK, with CT abdomen showing pancreatitis	Anabolic–androgenic steroid called “Guerilla Warfare”	1 month	Not performed	Clinical improvement with fluid hydration and pain management
Garg [17]	28-year-old male	Elevated amylase and lipase, with ultrasonography abdomen showing pancreatitis	Anabolic–androgenic steroid	Data unavailable	Not performed	Patient died
Rosenfeld *et al*. [28]	50-year-old male	Elevated lipase and triglycerides, with CT abdomen showing pancreatitis	Methandrostenolone	2 months	Not performed	Supportive therapy
Schäfer *et al*. [29]	26-year-old male	Hypercalcemia and elevated lactate dehydrogenase and pancreatic amylase, with CT abdomen showing pancreatitis	Oxymetholone, nandrolone decanoate, testosterone, epitestosterone, and erythropoietin	After second annual injection cycle	Not performed	Aggressive intravenous rehydration, transferred to intensive care unit because of anuria
Samaha *et al*. [2]	24-year-old male	Leukocytosis with left shift, hypercalcemia, and elevated amylase, lipase, and CPK	Testosterone	2 months	Not performed	Slow clinical improvement with fluid hydration and pain management
Rutten *et al*. [21]	40-year-old male	Elevated amylase and CRP with abdominal ultrasonography	Growth hormone	2 weeks	Not performed	Conservative treatment
Saka *et al*. [15]	16-year-old male	Elevated amylase and lipase with CT abdomen showing pancreatitis	l-Arginine and zinc	5 months	Not performed	Clinical improvement with ciprofloxacin, fluids, and bowel rest

The exact mechanism of how these drugs induce pancreatitis remains unknown, but postulated theories have included pancreatic duct constriction with localized angioedema and arteriolar thrombosis, hypersensitivity reactions and cytotoxic and metabolic effects [12]. The exact effects of AAS [20] and growth hormone [21] on DIAP are not well understood. Predictions are that AAS can induce an immune-mediated inflammatory response, direct cellular toxicity, pancreatic ductal constriction, arteriolar thrombosis, and metabolic effects in the pancreas [20], and it is observed in pediatric studies that growth hormone causes secretion of pancreatic enzymes [22]. Animal studies have shown that arginine, which is a potent secretagog of growth hormone, causes direct damage to the pancreatic acinar cells and dose-related necrotizing pancreatitis in rats [15].

Among the young and athlete population, performance-enhancing drugs (PED), most notably AAS, carry a significant risk of harmful side effects. Along with aesthetic and athletic benefits such as increased muscle mass, documented adverse effects include myocardial infarction [23], liver injury, kidney dysfunction, testicular atrophy, gynecomastia, and acne [16]. Growth hormone is another drug that is used by approximately 5% of US high-school students with doses up to 20 times the therapeutic level and commonly consumed as an adjunct to AAS in cycles of 4–6 weeks [24]. However, prior case reports on growth-hormone-induced acute pancreatitis are from growth-hormone-deficient children on treatment [25]. In addition to literature search on growth hormone, we found only one report on an animal study on the effects of clenbuterol on the pancreas. Guilhermo *et al.* have reported on the performance-enhancing effect of the acute administration of clenbuterol in horses and increased insulin secretion of pancreatic beta-2 adrenergic receptors [26]. However, this would not explain the occurrence of pancreatitis in mans. Overall, the mechanisms of pathology caused by PEDs, such as anabolic steroids, are better understood in the liver and kidney than the pancreas [1].

In our patient, magnetic resonance cholangiopancreatography (MRCP) and endoscopic ultrasound were not conducted to investigate for pancreatic divisum and occult microlithiasis, respectively, because of the rapid response to therapy, lack of history of a previous attack, and negative CT and ultrasound imaging. The patient was followed up for 1 year with no recurrent attacks and in the absence of drug use. The patient was not rechallenged because of the potential risks of recurrent pancreatitis. Of note is the lack of testing for genetic susceptibility for pancreatitis and to rule out familial forms of pancreatitis in most prior reports of drug-induced acute pancreatitis. Since the implication of specific drugs as a cause of acute pancreatitis is a diagnosis of exclusion, these rare genetic causes of pancreatitis must be ruled out before implicating the drug, as we have done in the current case.

## Conclusion

Athletes and bodybuilders risk using unknown or unregulated substances without medical advice that can harm them through mechanisms not well understood. Among these are “drug cocktails” such as the multiple AAS, clenbuterol, and growth hormone regimen that appears to have caused acute pancreatitis in our patient. Since this is the first case report on the potential toxicity of fluoxymesterone, mesterolone, drostanolone propionate, stanozolol, testosterone enanthate, testosterone propionate, and clenbuterol to the pancreas, we stress that further research is required to understand the mechanism of this combination of drugs in causing DIAP. Users must be aware of the potentially dangerous side effects of uncontrolled consumption and purchasing of such supplements and medications from unknown suppliers, and clinicians should be alert to the possibility of drug-induced pancreatitis in bodybuilders and other athletes.

## Data Availability

Not applicable.

## Abbreviations

DIAP: Drug-induced acute pancreatitis
CT: Computed tomography
ERCP: Endoscopic retrograde cholangiopancreatography
PED: Performance-enhancing drugs
WBC: White blood cell count
CRP: C-reactive protein
AST: Aspartate aminotransferase
ALT: Alanine aminotransferase
AP: Alkaline phosphatase
CPK: Creatine protein kinase
GGT: Gamma glutamyl transferase
LDH: Lactate dehydrogenase
CK: Creatine kinase
